# Vision System Measuring the Position of an Aircraft in Relation to the Runway during Landing Approach

**DOI:** 10.3390/s23031560

**Published:** 2023-02-01

**Authors:** Damian Kordos, Paweł Krzaczkowski, Paweł Rzucidło, Zbigniew Gomółka, Ewa Zesławska, Bogusław Twaróg

**Affiliations:** 1 Department of Avionics and Control Systems, Faculty of Mechanical Engineering and Aeronautics, Rzeszow University of Technology, al. Powstancow Warszawy 12, 35-959 Rzeszow, Poland; 2 College of Natural Sciences, University of Rzeszow, Pigonia St. 1, 35-959 Rzeszow, Poland

**Keywords:** automatic landing, neural network, aircraft position measurement, vision system

## Abstract

This paper presents a vision system that measures the position of an aircraft relative to the runway (RWY) during a landing approach. It was assumed that all the information necessary for a correct approach was based entirely on an analysis of the image of the runway and its surroundings. It was assumed that the way the algorithm works, as well as possible, should imitate the pilot’s perception of the runway. Taking into account the above and the fact that the infrastructure at each airport is different, it has been decided to use artificial neural networks with a dedicated learning process for any airport, based on the simulation environments. Such an action will enable the generation of a synthetic video sequence without the need for costly and time-consuming flights. The presented solution was tested in real flight conditions on an experimental aircraft, and the selected test results are presented in this article.

## 1. Introduction

The development of unmanned systems will require the use of the ground infrastructure currently used by manned aviation in the near future. Such action is essential, taking into account the exponentially growing demand for unmanned systems, primarily in the MALE (medium altitude long endurance) and HALE (high altitude long endurance) categories. Such a scenario is already envisaged, for example, through the introduction of this topic in large European projects such as ERA (enhanced RPAS automation) [1]. For many years, a lot of work has been carried out to improve the safety systems in aviation. A good example of such a product is the SOFIA (Safe Automatic Flight Back and Landing of Aircraft) [2] program, where the possibility of autonomous landing has been tested. In all aspects of a flight, one of the main and fundamental maneuvers is landing. This is the most difficult and dangerous part of the entire flight. Adequate infrastructure and good crew training are essential for a proper approach to landing. In the case of unmanned aerial vehicles (UAVs), this topic is extremely important. The correct landing approach is related to the equipment of the UAV itself and also to the airport infrastructure, which should be equipped with systems that allow for an automatic landing approach. The previously mentioned types of unmanned objects are already prepared to use manned aviation infrastructure based on the ILS (instrument landing system), the MLS (microwave landing system) or the GBAS (ground based augmentation system). However, a large number of airports do not have the infrastructure around them to enable landings using radio navigation instruments. Therefore, it is necessary to implement systems that can acquire the information necessary for the final phase of the flight, which is the landing. This shows how crucial and timely the drive is to develop new systems to support unmanned flights. This paper presents a vision system to measure the position relative to the runway during the approach to landing. These topics have been addressed by numerous researchers [3,4,5,6,7,8,9,10,11,12,13,14]. The runway detection process itself, based on color and features, is described in the article “Robust Method to Detect and Track the Runway during Aircraft Landing Using Color Segmentation and Runway Features” [15]. However, the method of extracting individual colors has its drawbacks such as a multitude of falsely recognized areas or susceptibility to errors due to changes in the lighting conditions (e.g., heavy cloud cover). Another method is to detect the edges of the runway, which are usually a clear transition between the colors. This type of system was proposed in the paper “A Robust Vision-Based Runway Detection and Tracking Algorithm for Automatic UAV Landing” [16]. In the article “Measurement of Aircraft Approach Using Airfield Image” [17], the proposed algorithm used the coordinates of the four corners of the runway to determine the position of the aircraft. When analyzing the literature describing the measuring of the aircraft position on the approach path, we could also distinguish methods where calculations were performed based on the vanishing point, formed by the longitudinal edges of the runway and the angles between the individual runway sides, as presented in the paper “Vision-Integrated Navigation System for Aircraft Final Approach in Case of GNSS/SBAS or ILS Failures” [18]. Another way to detect the runway was to use machine learning algorithms. The paper “Runway Detection and Localization in Aerial Images Using Deep Learning” [19] used neural networks to detect the runway area in images using Mask R-CNN (region-based convolutional neural networks) segmentation. The advantage of this method is the greater resistance to changing lighting conditions and the relative ease of further implementation. The disadvantage of this approach, on the other hand, is the need for a large amount of data to teach the neural network. This paper presents a method for measuring the position of an aircraft relative to the runway based on artificial neural networks (ANN) analyzing camera images. The measurement result of the proposed vision system should be the distance from the runway, the altitude above the runway, and the lateral deviation from the runway centerline. The proposed measured data should enable for a correct approach to landing. Due to the need to provide a large amount of learning data for ANNs, the following section describes the data acquisition process, which was based on simulation environments. For aerospace systems, reducing or eliminating the number of flights to acquire ANN learning material is crucial, often due to the technical feasibility of numerous flights and their high cost. The proposed solution was tested during real flights on the MP02 Czajka aircraft at the Rzeszow University of Technology EPRJ airport.

## 2. Structure of the Measurement Data Acquisition System

**Figure 1:**
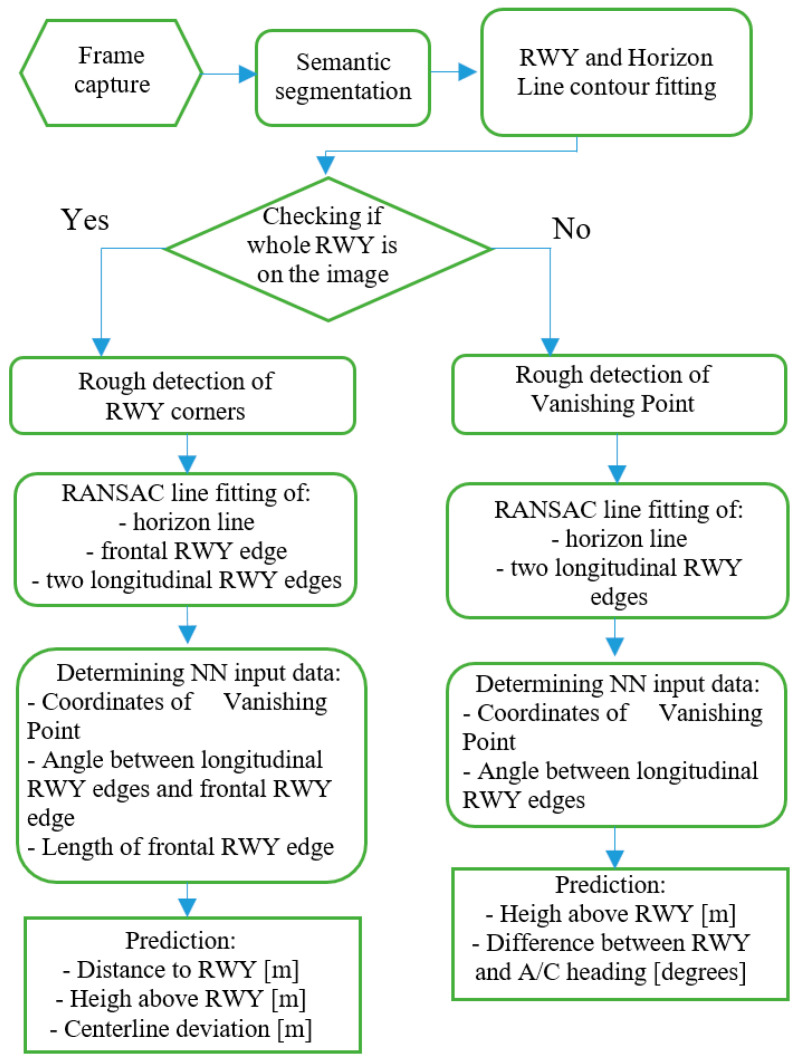
Operation of the system.

As mentioned, the proposed system was based on a system that processes a video image acquired from a camera placed on the aircraft and oriented along the line of symmetry, in the direction of flight. The operation of the measurement data acquisition process has several stages. A flow chart illustrating the operation of the system is shown in Figure 1.

**Figure 2:**
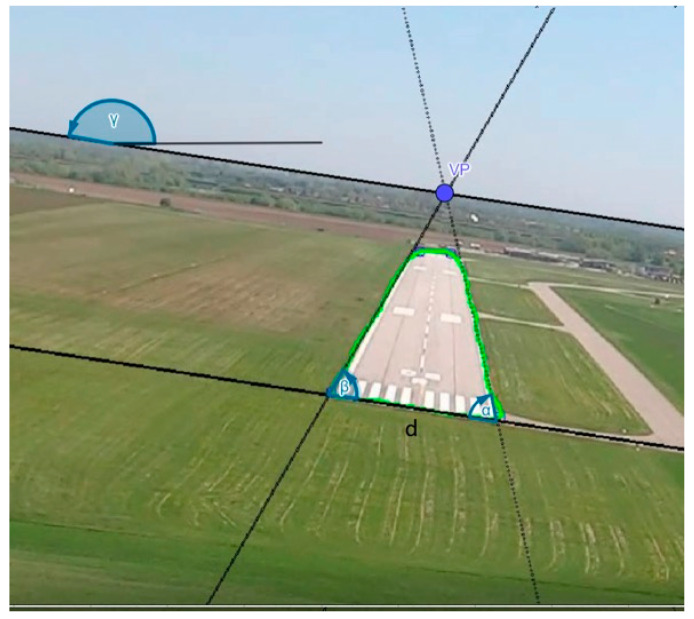
The detected lines were matched using the RANSAC algorithm.

The first step of the system is the registration of an image. This image is the input of semantic segmentation to recognize three different classes, being the runway, terrain, and the area above the horizon line. In the next step, the contours are adjusted to the highlighted areas. At this stage, the region of the intended runway is selected and a preliminary check is made to ensure that the aircraft is not directly above the runway. Here, the algorithm branches out, which is directly related to the differences between the approach to the runway and the final landing phase when the aircraft is already over the runway. The next step is to preliminarily analyze the runway area and measure the runway edge points and the vanishing point (VP), which is where the lateral runway edges converge. At the same time, the horizon line is defined. All the lines to be detected were matched using the RANSAC algorithm (Figure 2).

Based on the edge of the runway and the horizon line, the parameters that are input to the neural network that measure the position of the aircraft are specified. On the basis of the collected data, the position of the aircraft is calculated. When the aircraft is directly above the runway, two parameters are measured.

- The altitude above the runway;
- The angle between the longitudinal axis of the aircraft and the course of the runway (traverse).

In the case of a runway approach, the distance from the runway threshold, the altitude above the runway, and the lateral deviation from the centerline are measured. This approach allows for measuring the current approach path of the aircraft.

Two neural networks are used to implement the task. The first is responsible for semantic segmentation, based on the U-Net framework, and has an encoder–decoder architecture. To extract more information during encoding, EfficientNet-b3 was used as the encoder. At the last stage of measurements, another ANN was used to calculate the output parameters of the aircraft during the landing approach. This was a basic unidirectional neural network with two hidden layers.

**Figure 3:**
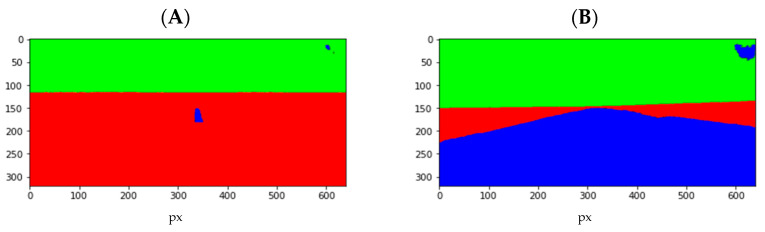
Example of runway segmentation (based on U-Net framework) at a long distance (**A**) and in its immediate vicinity (**B**).

The choice of U-Net for segmentation resulted from the analysis of several different models. One of the evaluation criteria was the ability of the neural network to segment the runway both at a long distance (Figure 3A) and in its immediate vicinity (Figure 3B) to use a single model for the entire flight. The U-Net network provided very stable results in the test data, with satisfactory processing speeds.

The networks were implemented using the Keras library and Tensorflow. The neural networks were trained using synthetically prepared data, as presented in the next section. The image resolution was reduced to 640 × 320, which ensured a measurement frequency of 14 Hz. The tests were carried out on a computer equipped with an Intel (R) Core (TM) i5-9300HF CPU @ 2.40 GHz processor and NVIDIA GeForce RTX 2060 graphics card.

## 3. Preparation of Learning Data and Training of ANN

Due to the nature of the system, the learning data should be individually prepared for each airport through numerous flight tests, the key elements of which would be the approaches including the landing itself. This involves large disparities in the infrastructure of the airport itself as well as in the surrounding immediate area. This solution is very costly due to the need to use aircraft of similar dimensions, the different dynamics of the approach depending on the size of the aircraft, and the costs associated with the landings (each landing, or so-called stopover—without losing speed before taking off again) imposed by the airport. Moreover, in addition to the direct costs, indirect costs arising from the airport’s workload must be taken into account, which is very important for large airports with flight operations every few minutes.

**Figure 4:**
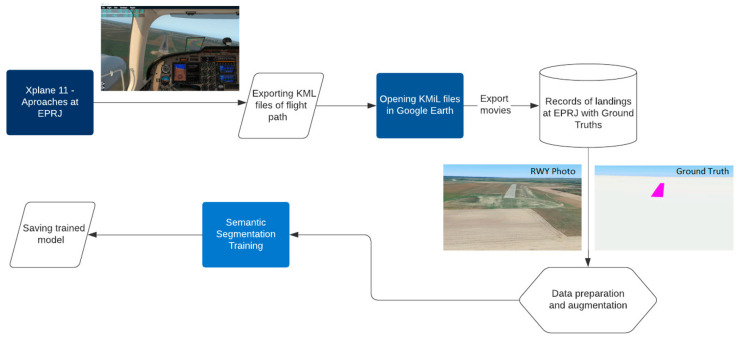
Proposed ANN learning methodology.

In order to avoid any inconvenience associated with the preparation of learning materials for the ANNs, the use of synthetic data for this purpose is proposed, which does not require landing approaches on real facilities. A similar approach to the problem of testing the vision system in the simulation environment was used, among others, in [20], the results of which were confronted with research in a real environment, obtaining satisfactory results [21]. Later in the paper, it will be demonstrated that synthetic data are capable of replacing material collected during flights. As part of the research, a methodology based on the simulation environment X-Plane 11 was developed [22], where approaches were performed and recorded for a specific runway (Figure 4). These flights are necessary to prepare a trajectory that will realistically reflect the behavior of the aircraft (simulation flight performed on a general aviation aircraft) during the approach to landing. It should be added that the proposed simulator has a very large and well-developed database of airports on all continents, which is not insignificant for the validity of the proposed solution.

The next step is to prepare the target data in video form. The data were generated using the Google Earth platform, which provides a detailed view of the entire world. This platform is continually being developed and the data are constantly updated, which also has an impact on the ANN training process [23]. Approximately 100,000 synthetic pictures of the runway with annotations were prepared. The pictures were divided into training and validation data (approximately 10%). Strong augmentation was used because of the use of synthetic data, significantly deviating from the target data. The annotations were created by applying a simple and uniform segmentation color (magenta) to the runway surface. Then, the recordings were exported with the original image of the runway and its marked (annotated) image.

Training of the neural network determining the position of the aircraft relative to the runway based on data from the vision system was carried out in several stages:

- Preparation of a mathematical model of the camera, a function that determines the view of the runway (vision parameters) on the camera image for the given position of the aircraft relative to the runway;
- Determining the parameters of the selected camera (focal length, principal point, etc.);
- Creation of the input–output data generator (runway vision parameters—aircraft position);
- Selection of the neural network (shallow neural network);
- Neural network training based on generator data;
- Tests of neural networks with different architects (number of hidden layers).

The U-Net neural network was used to determine the vision parameters of the runway (such as the angles between the individual edges of the runway) that constitute the input of the basic unidirectional neural network with two hidden layers. At its exit, we obtain the position of the aircraft relative to the runway (distance from the runway, height above the runway, linear deviation from the central line, angle between the axis of the runway, and the longitudinal axis of the aircraft).

To investigate the feasibility of using synthetic data to train ANNs, a flight was conducted in the X-Plane 11 environment. The flights were carried out on a General Aviation (GA) aircraft, while the landing site was the Rzeszów University of Technology Airport (EPRJ). The ANN prepared on the basis of the developed materials was verified during flights on a real object, also in GA class.

## 4. Test Object and Test Flight Scenario

**Figure 5:**
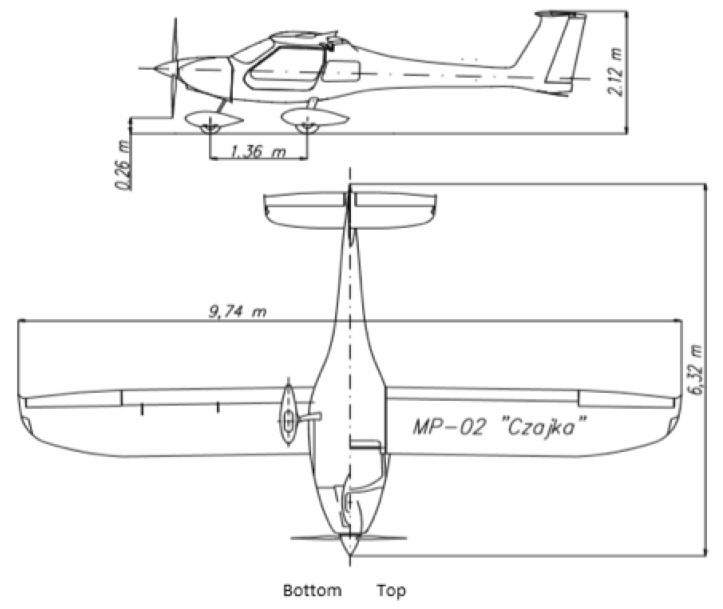
Outline of the MP-02 Czajka plane with dimensions.

**Figure 6:**
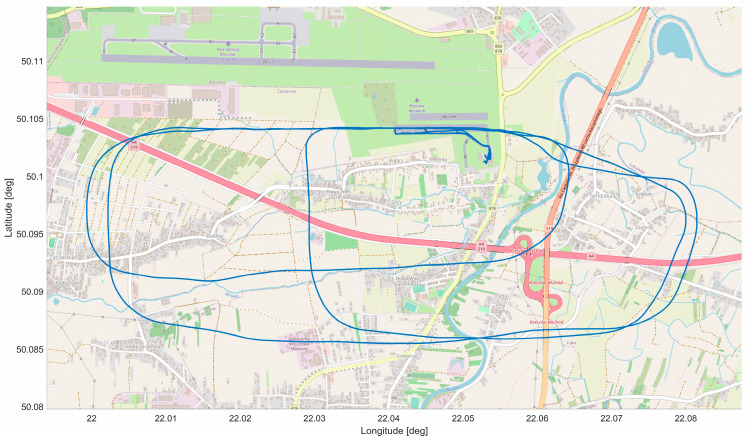
View of the research flight path.

**Figure 7:**
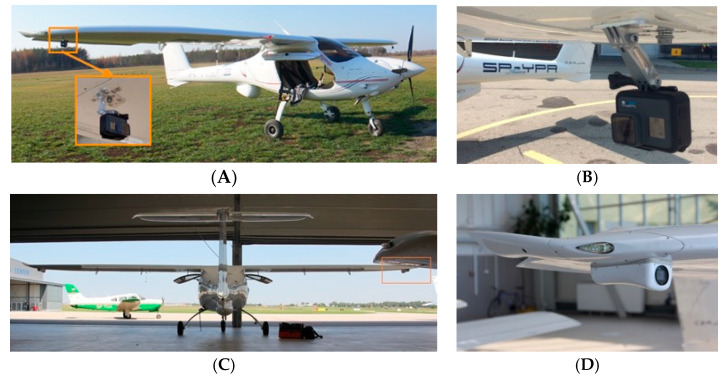
MP-02 Czajka plane with a GoPro 7 camera installed for preliminary experiments (**A**,**B**) and its installation for the planning of more advanced experiments in the future (**C**,**D**).

In-flight tests were carried out on the Czajka MP-02A aircraft (Figure 5), which is registered in the SPECIAL class, as a research aircraft in the Department of Avionics and Control Systems at the Rzeszów University of Technology. The verification flight plan included three flights at EPRJ Airport and is shown in Figure 6. The flights performed were intended to differ from each other so that the different approach paths would display slightly different parameters. The video recording device used for the tests was a GoPro 7 sports camera. This was placed directly under the right wing of the aircraft (Figure 7). This camera attachment provides a very good picture of the runway during the approach to landing, but it does affect the indication of the aircraft’s position relative to the runway axis. Videos were recorded at 25 frames per second with full HD resolution. The reference data for the vision system were taken from the onboard recorder (Dynon Avionics D700 + SV-ADAHRS + SV-GPS) and are the barometric altitude, position determined by the GNSS system, and magnetic heading from the AHRS system.

## 5. Test on a Real Object

**Figure 8:**
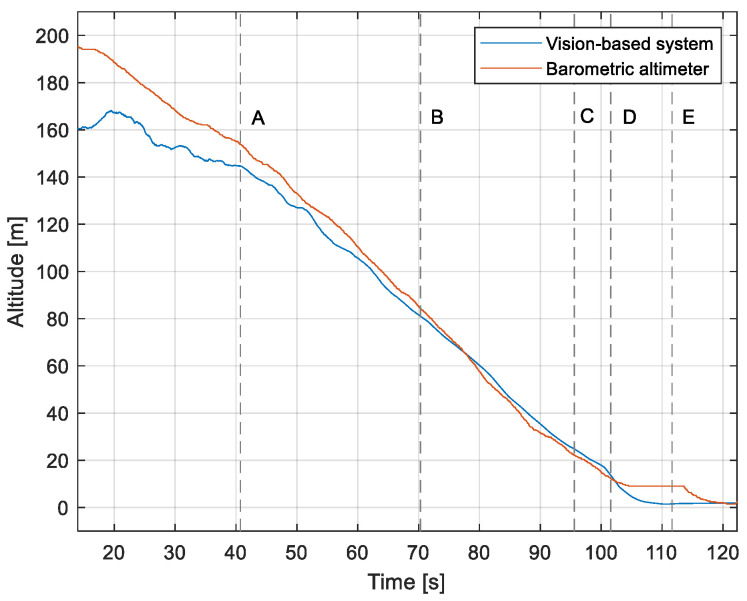
Altitude relative to the airside: Flight no. 1 (markers A–E correspond to the moment of recording the images in Figure 9).

**Figure 9:**
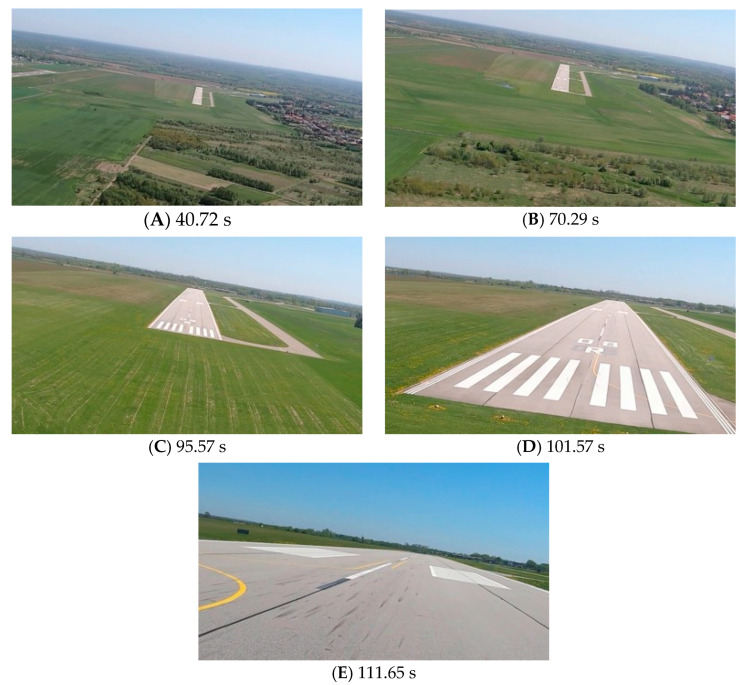
Recorded images related to the altitude measurements presented in Figure 8.

**Figure 10:**
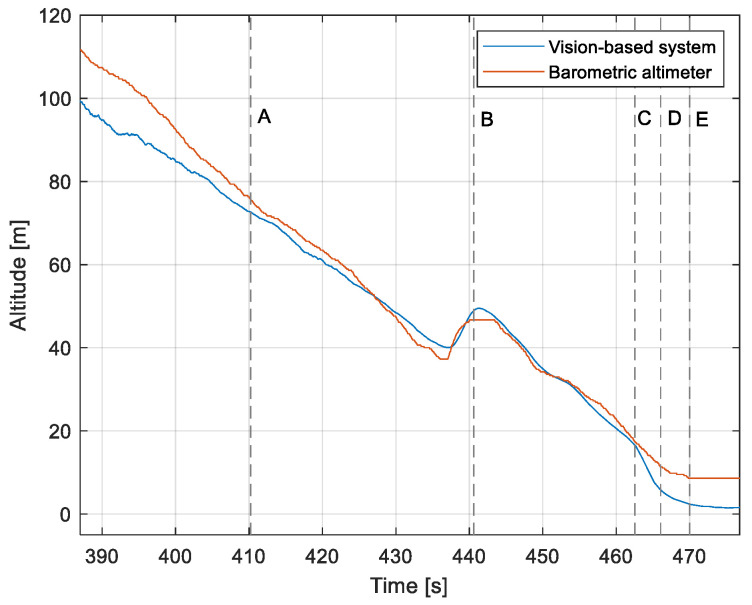
Altitude relative to the airside: Flight no. 2 (markers A–E correspond to the moment of recording the images in Figure 11).

**Figure 11:**
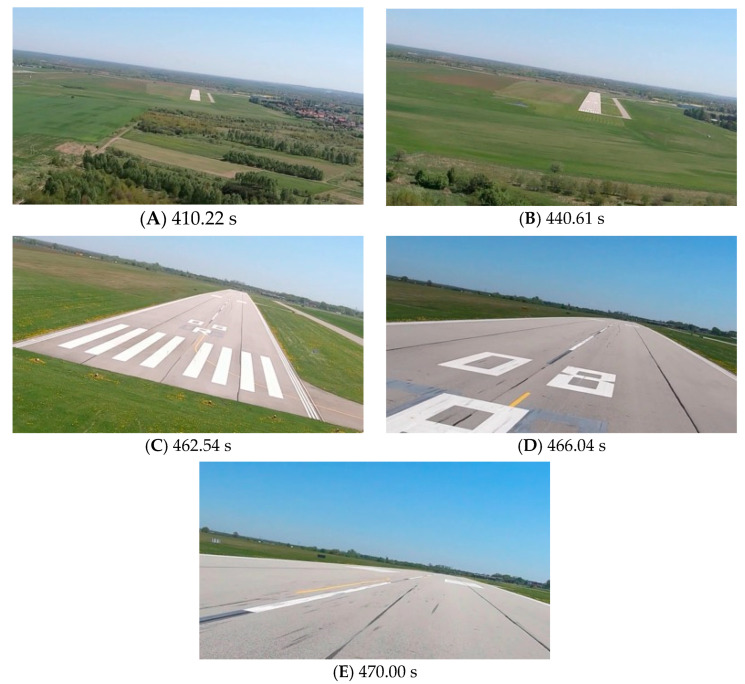
Recorded images related to the altitude measurements presented in Figure 10.

**Figure 12:**
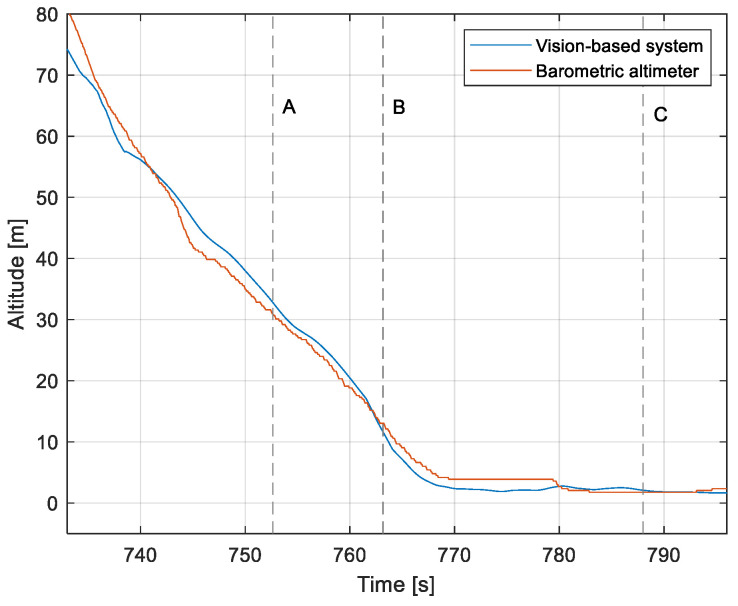
Altitude relative to the airside: Flight no. 3 (markers A–C correspond to the moment of recording the images in Figure 13).

**Figure 13:**
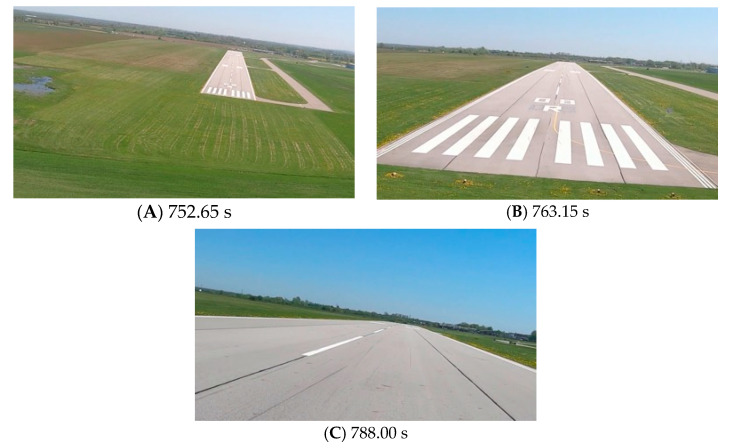
Recorded images related to the altitude measurements presented in Figure 12.

**Figure 14:**
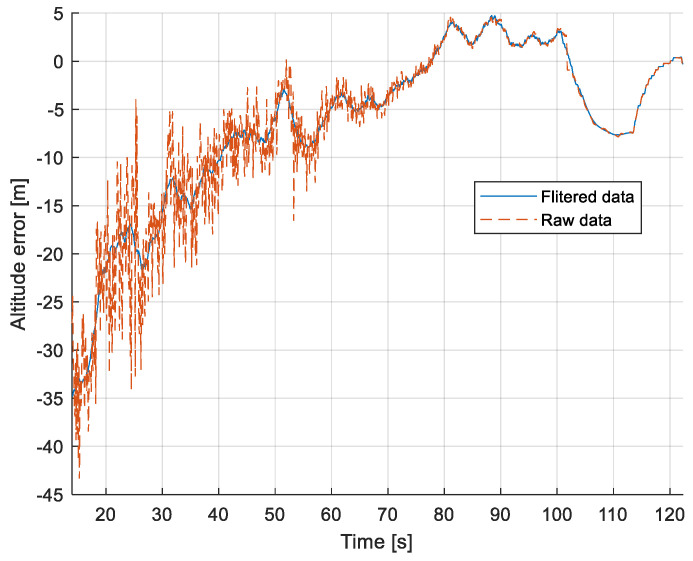
Relative error of altitude measurement using the vision method in relation to the barometric altitude: Flight no. 1.

**Figure 15:**
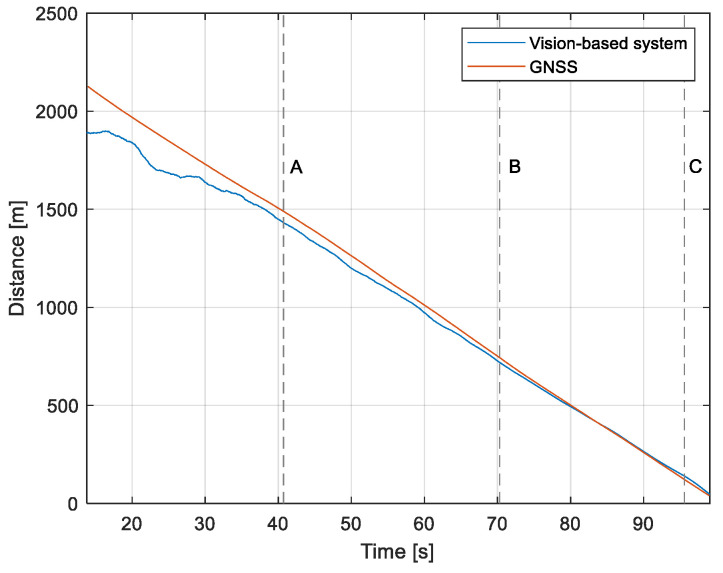
Distance from the runway: Flight no. 1 (markers A–C correspond to the moment of recording the images in Figure 9).

**Figure 16:**
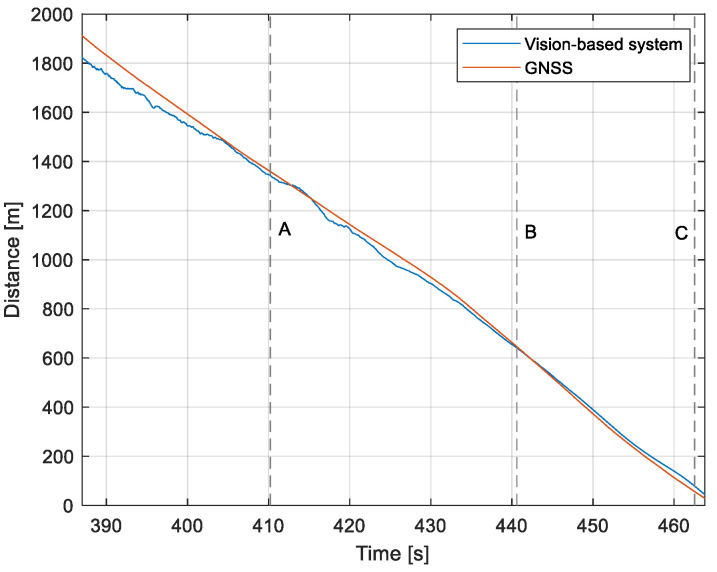
Distance from the runway: Flight no. 2 (markers A–C correspond to the moment of recording the images in Figure 11.

**Figure 17:**
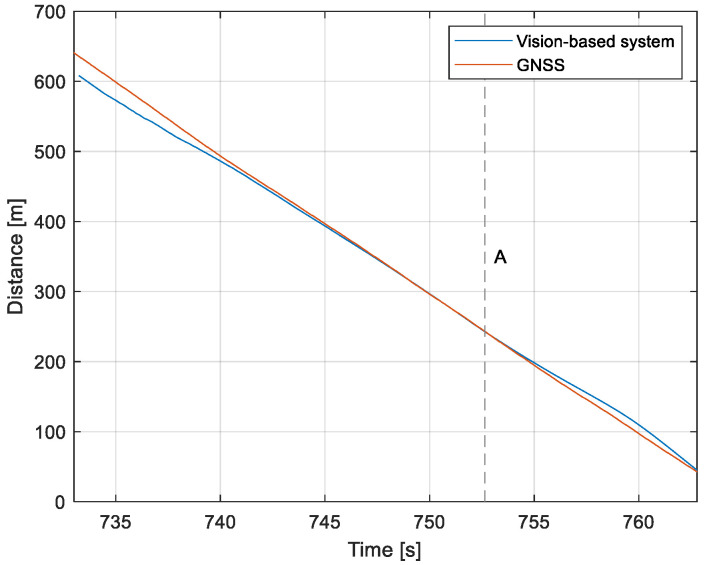
Distance from the runway: Flight no. 3 (marker A corresponds to the moment of recording the images in Figure 13.

**Figure 18:**
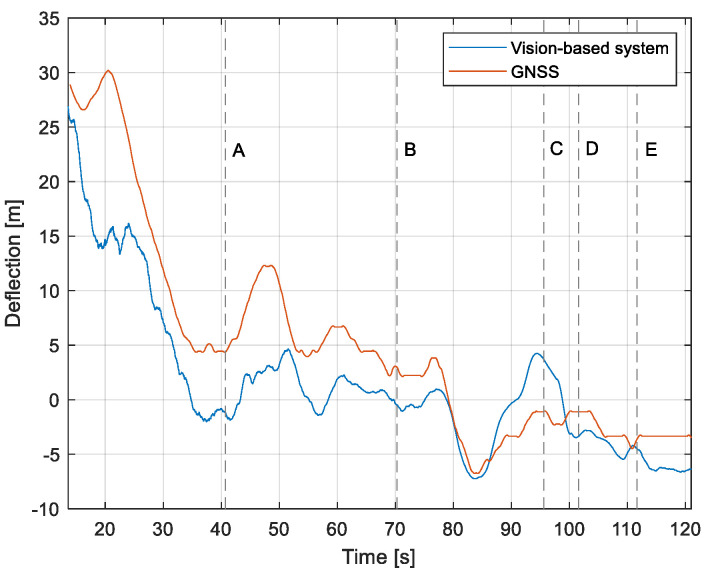
Linear lateral deflection from the center line: Flight no. 1 (markers A–E correspond to the moment of recording the images in Figure 9).

**Figure 19:**
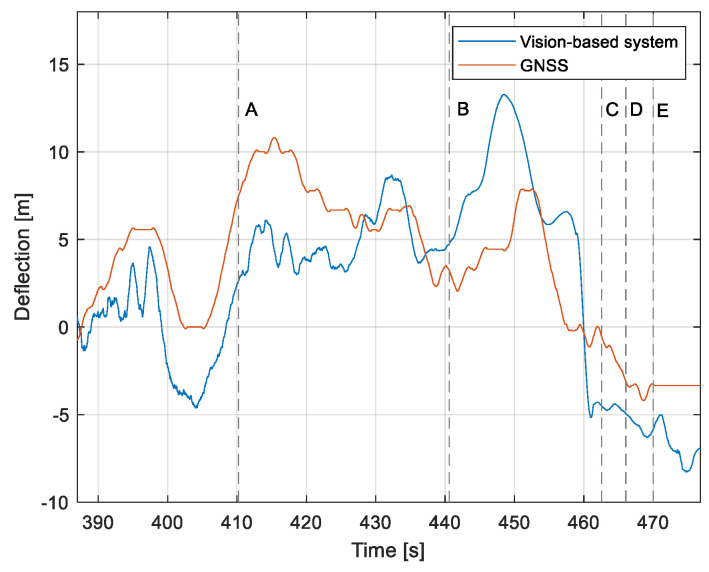
Linear lateral deflection from the center line: Flight no. 2 (markers A–E correspond to the moment of recording the images in Figure 11.

**Figure 20:**
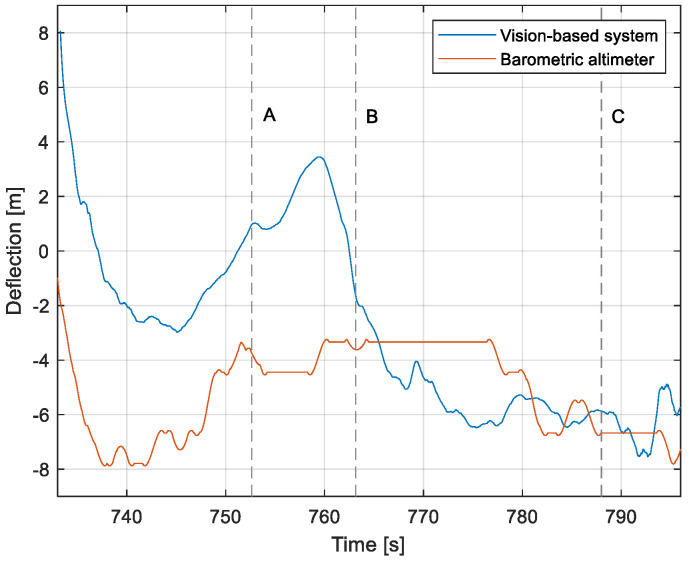
Linear lateral deflection from the center line: Flight no. 3 (markers A–C correspond to the moment of recording the images in Figure 13.

**Figure 21:**
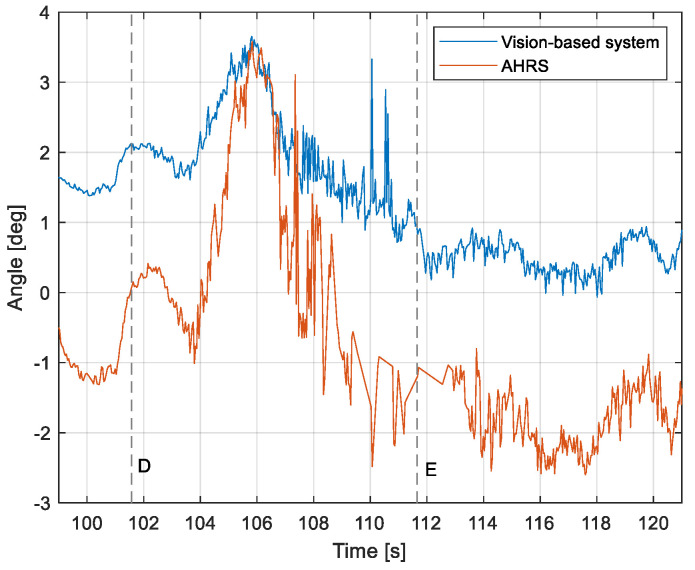
Angle between the longitudinal axis of the aircraft and the centerline: Flight no. 1 (markers D,E correspond to the moment of recording the images in Figure 9).

**Figure 22:**
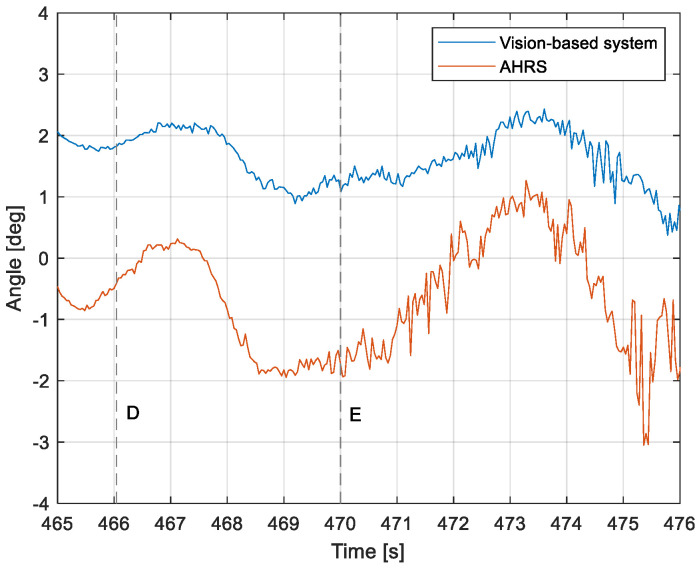
Angle between the longitudinal axis of the aircraft and the centerline: Flight no. 2 (markers D,E correspond to the moment of recording the images in Figure 11.

**Figure 23:**
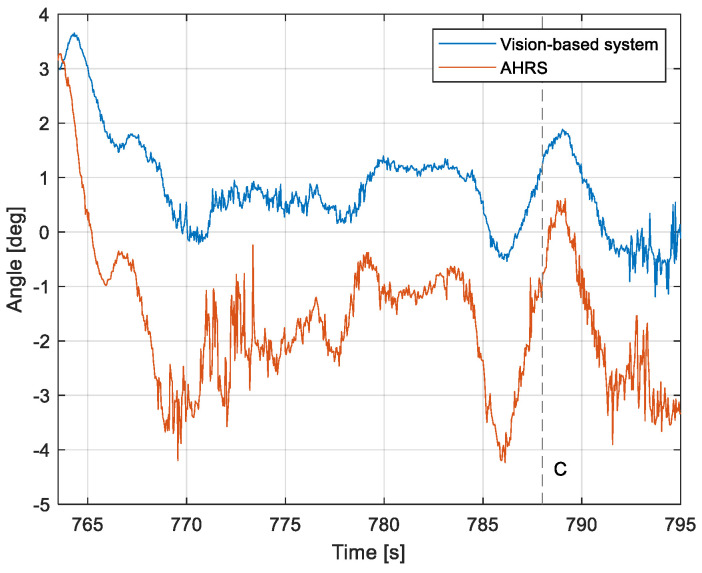
Angle between the longitudinal axis of the aircraft and the centerline: Flight no. 3 (marker C corresponds to the moment of recording the images in Figure 13).

The purpose of the tests carried out was to check how the developed system worked under real conditions. The measured quantities were the altitude above the runway, the runway distance, and the lateral deviation from the centerline. As the aircraft passed the runway threshold, the altitude and angle between the runway direction and the longitudinal axis of the aircraft were calculated. These values allowed us to determine the spatial position on the approach path, while over the runway themselves, they provide information about the landing flare altitude and traverse. The reference distance from the runway and the lateral deviation from the centerline were determined using data from the satellite navigation system. The altitude of the aircraft above the EPRJ runway was measured using a barometric altimeter [24]. A distinctive, easily recognizable point was used to synchronize the time of the camera and the on-board recorder. A moment of rapid heading change was chosen when leaving the runway. Due to the nature of the measurement system, when the aircraft is far from the runway, the runway image occupies only a small part of the image area. For this reason, the measured parameters had a relatively large measurement uncertainty. To compare the quantities measured by the proposed system with the data recorded by the on-board measuring systems, trend lines were established for the course of each of the waveforms obtained by the tested system. For this purpose, a Savitzky–Golay filter was used. In their work, they proposed a method for smoothing high-noise data [25]. In the following section, the results for the individual flight parameters are presented. Research flights were carried out on 12 May 2021 at the Rzeszów-Jasionka EPRJ Airport, in the morning hours. CAVOK weather conditions, temperature of 15 °C, wind from direction 110 at 20 km/h [26]. The measured data values obtained with the proposed vision system are shown in Figure 8, Figure 9, Figure 10, Figure 11, Figure 12, Figure 13, Figure 14, Figure 15, Figure 16, Figure 17, Figure 18, Figure 19, Figure 20, Figure 21, Figure 22 and Figure 23. The solid line shows the trend line fitted to the data obtained. When the graph was analyzed, it was observed that the results of each attempt differed from each other. The duration of the approaches in attempts one and two was significantly longer than the approach time on flight three. This was related to the nature of the flight made, where the final landing had the steepest path. It can also be noted that the accuracy of the results obtained decreased significantly with an increasing distance from the runway, which is a characteristic of this type of measurement system. Furthermore, to illustrate the accuracy of the measurement, the results of the vision system were compared with the traditional measurement system installed on board the aircraft. On this basis, the accuracy of the proposed system can be estimated. The graphs from the on-board systems are shown in orange, while those from the vision system are shown in blue. Figure 8, Figure 10 and Figure 12 show the altitude of the aircraft over the runway for three landing approaches.

By analyzing the graphs of the results obtained, it can be seen that the system provided satisfactory results and the correctness of the results obtained with the proposed vision system was confirmed by comparative data. It can be seen that the time waveforms of the quantities measured by the vision system such as the distance from the runway (Figure 15, Figure 16 and Figure 17) and the altitude above the runway (Figure 8, Figure 10 and Figure 12) were very closely in line with the satellite navigation and barometric altimeter data. Note the discontinuities that appeared when measuring the altitude above the runway, which were the result of a change in the altitude calculation algorithm. This is something that can be easily eliminated, but for the purposes of the verification of the proposed method itself, it is of little importance.

Figure 9, Figure 11 and Figure 13 show selected frames from the recorded and analyzed video recordings. Figure 9A–C, Figure 11A,B and Figure 13A show situations in which the runway is visible from a relatively long distance and occupies only a fragment of the frame. By referring to the appropriate markers (indicated by a dotted line) in Figure 8, Figure 10 and Figure 12, it is easy to determine the time of measuring the altitude to which they correspond. In the case of flight no. 1, an analysis of the relative error of the altitude measurement (calculated relative to the barometric altitude measurement) was also included, which presents the raw measurement data obtained at the ANN output and the filtered (resulting) data.

Determination of the distance from the runway threshold using the visual method is possible only when it is fully visible in the image. Referring Figure 9A–C, Figure 11A–C and Figure 13A to the runway distance plots (Figure 15, Figure 16 and Figure 17), we noted that the runway distance calculation function only worked until the entire runway was visible in the frame. For this reason, the time axis in Figure 15, Figure 16 and Figure 17 was limited to the range where the distance calculation was still possible. For the same reason, the time axis was limited in the graphs illustrating the calculation of linear lateral deviation (Figure 18, Figure 19 and Figure 20) and the angle between the longitudinal axis of the aircraft and the centerline (Figure 21, Figure 22 and Figure 23).

When measuring the lateral deviation from the runway axis (Figure 18, Figure 19 and Figure 20), in flights 1 and 2, we could see a cross between the GNSS graphs and the vision system, which was caused by not placing the camera in the axis of the aircraft, or flying with a traverse. This will be more significant at long distances, but as we get closer to the runway, it is of little significance, as we can see from the graphs. The difference between the runway distance plots was approximately 5 m to the runway, which was due to the placement of the camera on the wingtip and the GPS module near the longitudinal axis of the aircraft (Figure 5 and Figure 7). Analyzing the altitude graphs over the airside (Figure 8, Figure 10 and Figure 12), we could see a constant difference between the measured altitudes of about 2 m, which was related to the camera being placed at a height of 1.7 m above the aircraft landing gear. The uncertainty of the quantities measured by the vision system is highly variable and largely depends on the position of the aircraft relative to the runway, and is inversely proportional to the size of the runway image in the camera image. When the plane is far from the airport, the runway is only a small part of the picture.

As we approach the runway, the image becomes clearer and larger, resulting in better performance of the altitude and distance estimation from the vision system (Figure 14). This situation continues until the runway outline begins to extend beyond the frame of the frame. Altitude estimation is then still performed, but it becomes impossible to determine the distance from the runway (however, this effect should be considered obvious and expected). Linear lateral deviation as well as height was determined by the vision system throughout the whole test (Figure 18, Figure 19 and Figure 20). The parameter that was the most difficult to determine by the vision system was the angle measured between the longitudinal axis of the aircraft (aircraft heading) and the centerline of the runway (runway heading). Determining it became possible only when the central line of the runway was very well visible in the frame (this corresponds to Figure 9D,E, Figure 11D,E and Figure 13C). These measurements related to the AHRS system (Figure 21, Figure 22 and Figure 23) indicate a similar work of both measurement systems in dynamic states and a difference in their measurements of 2–2.5 deg for each of the three flights. This difference is most likely due to an imperfect camera installation.

The testing carried out on the real object allowed for a preliminary check of the accuracy of the proposed system. It can be assumed that the vision system fulfils its purpose well; however, the error it exhibits still needs to be thoroughly verified with more tests. The operation of the system directly above the runway is also very promising. At short distances from the runway, the vision system had a similar accuracy to classical on-board instruments, which is crucial in the landing approaches.

## 6. Conclusions

The proposed system for measuring the position of the aircraft during approach to landing uses semantic segmentation and image processing algorithms to determine parameters that characterize the geometry of the runway image. The runway geometry data collected in the image is the input of a neural network that determines the position of the aircraft on the approach path. The machine learning process was carried out on the basis of synthetic data. In the case of semantic segmentation, the images that are part of the learning set were developed using X-Plane 11 and Google Earth. A neural network was created to determine the spatial position of the aircraft on the basis of the runway geometry using a mathematical model of a GoPro 7 camera and an image of the runway of the EPRJ Airport.

The operation of the proposed system was checked on the basis of test flights with the MP-02 ‘Czajka’ aircraft. Three approaches were made to the runway of the EPRJ Airport. The videos recorded were used to check how the developed system performed in real flight conditions and whether ANN training on synthetic data, acquired according to the proposed method, will enable correct training of the measurement network. The results of all of the measured quantities are presented in the relevant graphs. The time courses of the results were compared with data from the satellite navigation system, the heading system, and the barometric altimeter. From the graphs, a significant similarity could be observed between the results obtained from the operation of the vision system and the data from the flight recorder of the aircraft. Moreover, the frequency of operation of the system, which was 14 Hz, is sufficient to control GA-class aircraft. This confirms the validity of the proposed method.

The results obtained after appropriate filtering could be used as a data source in the automatic landing process, as the authors wish to demonstrate in further work.

## Data Availability

The data presented in this study are available on request from the corresponding author.
